# Association of body fat percentage with diabetes in hypertensive adults of different genders: a cross-sectional study

**DOI:** 10.3389/fendo.2025.1467886

**Published:** 2025-03-04

**Authors:** Jingan Rao, Congcong Ding, Yumeng Shi, Wei Zhou, Chao Yu, Tao Wang, Lingjuan Zhu, Xiao Huang, Huihui Bao, Xiaoshu Cheng

**Affiliations:** ^1^ Department of Cardiovascular Medicine, The Second Affiliated Hospital, Jiangxi Medical College, Nanchang University, Nanchang, Jiangxi, China; ^2^ Jiangxi Provincial Cardiovascular Disease Clinical Medical Research Center, Nanchang, Jiangxi, China; ^3^ Jiangxi Sub-Center of National Clinical Research Center for Cardiovascular Diseases, Nanchang, Jiangxi, China; ^4^ Center for Prevention and Treatment of Cardiovascular Diseases, The Second Affiliated Hospital, Jiangxi Medical College, Nanchang University, Nanchang, Jiangxi, China

**Keywords:** body fat percentage, diabetes, gender difference, hypertension, cohort study

## Abstract

**Background:**

While numerous epidemiological studies on body fat and diabetes already exist, there remains a scarcity of evidence regarding gender differences within hypertensive populations. The aim of this study was to examine gender-specific differences in the association of body fat percentage (BFP) with diabetes.

**Methods and results:**

This cross-sectional study encompassed 14,228 hypertensive patients from the Chinese Hypertension Registry. An easily obtainable anthropometric parameter, Clínica University de Navarra-Body Adiposity Estimator (CUN-BAE) equation was used to calculate body fat percentage (BFP). Diabetes was defined as the self-report of a previous diagnosis of diabetes, fasting blood glucose ≥ 7.0mmol/l, and the use of antidiabetic agents. The average BFP was 24.5% in men and 37.0% in women. Multivariate logistic regression analysis revealed a dose-dependent relationship between BFP and the risk of diabetes in men (odds ratio [OR] 1.09, 95% CI 1.07, 1.11) and women (OR 1.06, 95% CI 1.04, 1.07) while considering BFP as a continuous variable. After taking BFP as the quartile across different genders, compared with Q1 group, the risk of diabetes in Q4 group increased 176% (OR 2.76, 95% CI 2.15, 3.55) in men and 66% (OR 1.66, 95% CI 1.36, 2.03) in women. Furthermore, the positive association was found to be more significant in men, whether BFP was considered a continuous variable (*P* for interaction = 0.016) or a categorical variable in quartiles (*P* for interaction = 0.008). In addition, the positive association between BFP and diabetes remained consistent across various subgroups.

**Conclusion:**

BFP is positively associated with the increased risk of diabetes in hypertensive population, especially in men.

## Introduction

Approximately 11.9% of Chinese adults are affected by diabetes, and this number is steadily increasing. However, in China, only 38.0% of diabetes cases are properly recognized, with 34.1% receiving treatment and 33.1% having their blood glucose levels effectively controlled (1). Hyperglycemia is recognized for triggering various vascular complications and increasing oxidative stress through multiple pathways (2). Atherosclerosis, a leading cause of mortality in diabetes patients, is primarily associated with natural and modified lipids (3–5). Furthermore, obesity, characterized by an excessive proportion of body fat, is closely linked to the risk of diabetes (6, 7). Research conducted about 20 years ago underscored body mass index (BMI) as an independent predictor of diabetes (8). Bariatric surgery has been identified as a promising method for achieving diabetes remission (9). In conclusion, obesity not only poses a significant risk for diabetes and cardiovascular disease but also contributes to premature mortality and disability in the population at large (10–12).

To accurately assess the complexity of obesity, there is a pressing need for more precise indicators. Although the BMI is widely utilized and easily accessible, its simplicity hinders its ability to capture the multifaceted nature of obesity. BMI relies solely on height and weight measurements, failing to differentiate between fat and muscle mass or account for the distribution of body fat. Recognizing the limitations of BMI, recent findings have challenged its effectiveness in predicting health risks, such as diabetes (13, 14). Research by Lu et al., involving 5860 Chinese participants, revealed that increased BMI alone, without abdominal obesity, did not significantly correlate with the risk of type 2 diabetes. Conversely, individuals with abdominal obesity experienced a 55% increase in the incidence of type 2 diabetes (15). Such limitations have prompted the exploration of alternative obesity indicators. Given the crucial role of fat accumulation in the pathogenesis of diabetes, it is imperative to explore a new fat index. Notably, body fat percentage (BFP) has demonstrated greater accuracy than BMI in measuring obesity, particularly central obesity, and has shown improved predictive capabilities for certain diseases (16, 17).

Previous studies have explored the relationship between BFP and diabetes risk under different backgrounds (18, 19). However, whether the relationship between them in patients with hypertension is the same as that of other populations is still rarely explored. Given the significant differences in BFP levels between men and women due to variations in sex hormones and body fat distribution, the association between BFP and diabetes has seldom been investigated across different genders. Therefore, we conducted a cross-sectional study to explore the association between BFP and diabetes risk and the gender differences in hypertensive population.

## Methods

### Study population

The protocol was obtained from the China H-type Hypertension Registry Study (registration number: ChiCTR1800017274). The trial design and methods have been previously introduced. This is a real-world observational study that was registered in August 2018 in China. Eligible subjects were hypertensive patients aged 18 and over. The exclusion criteria for this study include the following: (1) Inability to provide informed consent due to psychological or nervous system damage. (2) Inability to comply with the study’s follow-up protocol or planning to relocate in the near future. (3) Patients evaluated by the investigator who were deemed unsuitable for inclusion or long-term follow-up. All participants provided written informed consent. But since our investigation is mainly conducted in rural areas, the majority of participants are aged between 50-70 years old (**Table 1**).

**Table 1:** Clinical characteristics of the study population according to body fat percentage among men and women.

Variable	Body fat percentage, %									
	Men				*P* value	Women				*P* value
	Q1 (<21.7)	Q2 (21.7-24.5)	Q3 (24.5-27.2)	Q4 (≥27.2)		Q1 (<34.3)	Q2 (34.3-37.0)	Q3 (37.0-39.9)	Q4 (≥39.9)	
N	1680	1679	1680	1680		1877	1877	1877	1878	
Age, year	64.5 ± 9.1	64.8 ± 9.9	63.6 ± 9.9	62.3 ± 10.1	<0.001	62.7 ± 9.4	64.3 ± 8.8	63.8 ± 8.9	64.4 ± 8.7	<0.001
BMI, kg/m^2^	19.5 ± 3.9	22.1 ± 1.4	24.3 ± 1.3	27.7 ± 2.4	<0.001	19.8 ± 1.8	22.5 ± 1.3	24.7 ± 1.2	28.3 ± 2.6	<0.001
Current smoking, n (%)	981 (58.4)	849 (50.6)	729 (43.4)	691 (41.1)	<0.001	137 (7.3)	106 (5.7)	96 (5.1)	72 (3.8)	<0.001
Current drinking, n (%)	688 (41.0)	692 (41.2)	670 (39.9)	628 (37.4)	0.091	84 (4.5)	110 (5.9)	100 (5.3)	93 (5.0)	0.263
Physical activity, n (%)					<0.001					<0.001
Mild	711 (50.0)	759 (55.5)	789 (57.7)	827 (59.7)		832 (55.1)	836 (55.9)	834 (55.6)	921 (62.5)	
Moderate	374 (26.3)	319 (23.3)	332 (24.3)	312 (22.5)		337 (22.3)	343 (22.9)	341 (22.8)	283 (19.2)	
Vigorous	337 (23.7)	289 (21.1)	246 (18.0)	246 (17.8)		342 (22.6)	316 (21.1)	324 (21.6)	269 (18.3)	
SBP, mmHg	145.6 ± 18.8	146.9 ± 18.3	145.7 ± 17.4	146.7 ± 17.3	0.052	150.1 ± 18.1	150.1 ± 17.5	150.4 ± 17.3	150.7 ± 17.3	0.706
DBP, mmHg	88.2 ± 11.0	89.7 ± 10.8	90.6 ± 11.0	92.2 ± 11.0	<0.001	87.3 ± 11.2	87.6 ± 10.2	88.2 ± 10.0	88.3 ± 10.1	0.010
Laboratory results										
TC, mmol/L	4.8 ± 1.0	4.9 ± 1.1	5.0 ± 1.1	5.0 ± 1.1	<0.001	5.3 ± 1.1	5.4 ± 1.1	5.3 ± 1.1	5.4 ± 1.2	0.002
TG, mmol/L	1.0 (0.8-1.3)	1.2 (0.9-1.8)	1.5 (1.0-2.2)	1.7 (1.2-2.6)	<0.001	1.3 (1.0-1.8)	1.6 (1.1-2.2)	1.7 (1.2-2.5)	1.8 (1.4-2.6)	<0.001
HDL-C, mmol/L	1.7 ± 0.5	1.6 ± 0.4	1.5 ± 0.4	1.4 ± 0.4	<0.001	1.7 ± 0.5	1.6 ± 0.4	1.5 ± 0.4	1.5 ± 0.4	<0.001
LDL-C, mmol/L	2.6 ± 0.7	2.8 ± 0.8	2.9 ± 0.8	3.0 ± 0.8	<0.001	3.0 ± 0.8	3.1 ± 0.8	3.1 ± 0.8	3.2 ± 0.8	<0.001
FPG, mmol/L	5.7 ± 1.1	6.0 ± 1.4	6.1 ± 1.5	6.5 ± 1.8	<0.001	6.1 ± 1.4	6.2 ± 1.6	6.4 ± 1.9	6.5 ± 1.9	<0.001
eGFR, mL/min/1.73 m^2^	85.6 ± 21.2	85.7 ± 20.2	85.9 ± 20.1	86.0 ± 20.3	0.934	92.1 ± 19.7	90.4 ± 19.3	90.3 ± 19.5	88.3 ± 20.4	<0.001
Hcy, μmol/L	16.8 (13.6-22.1)	16.7 (13.7-21.9)	16.6 (13.5-21.4)	16.2 (13.4-20.9)	0.292	13.7 (11.7-16.8)	13.9 (11.9-17.1)	13.7 (11.8-16.9)	14.0 (12.0-17.2)	0.163
History of disease, n (%)										
Stroke	146 (8.7)	150 (8.9)	146 (8.7)	127 (7.6)	0.480	107 (5.7)	95 (5.1)	98 (5.2)	113 (6.0)	0.554
CHD	83 (4.9)	83 (4.9)	101 (6.0)	102 (6.1)	0.270	80 (4.3)	96 (5.1)	91 (4.9)	93 (5.0)	0.637
Diabetes mellitus^$^	132 (7.9)	221 (13.2)	295 (17.6)	430 (25.6)	<0.001	270 (14.4)	346 (18.4)	432 (23.0)	491 (26.1)	<0.001
Medication use, n (%)										
Antihypertensive drugs	1016 (60.5)	1062 (63.3)	1115 (66.4)	1151 (68.5)	<0.001	1084 (57.8)	1194 (63.7)	1291 (68.8)	1311 (69.9)	<0.001
Lipoprotein- lowering drugs	37 (2.2)	55 (3.3)	57 (3.4)	88 (5.2)	<0.001	38 (2.0)	59 (3.1)	76 (4.1)	96 (5.1)	<0.001
Glucose-lowering drugs	29 (1.7)	58 (3.5)	75 (4.5)	129 (7.7)	<0.001	59 (3.1)	105 (5.6)	143 (7.6)	157 (8.4)	<0.001

Data with normal distribution are expressed as mean ± SD or median (interquartile range) and numbers (percentage) as appropriate.

BMI, body mass index; SBP, systolic blood pressure; DBP, diastolic blood pressure; Hcy, homocysteine; FPG: fasting plasma glucose; TC, serum total cholesterol; TG, triglycerides; HDL-C, high-density lipoprotein cholesterol; LDL-C, low-density lipoprotein cholesterol; eGFR, estimated glomerular filtration rate; CHD, coronary heart disease.

^$^diabetes mellitus was defined as self-reported physician diagnosis of diabetes or FBG concentration ≥7.0 mmol/L or use of glucose-lowering drugs.

This study was conducted in accordance with the Declaration of Helsinki and approved by the Ethics Committee of the Institute of Biomedical Sciences, Anhui Medical University (Ethics NO. CH1059), and the Second Affiliated Hospital of Nanchang University (Ethics NO. 2018019). All patients provided signed informed consent before participating in this study.

**Figure 1:**
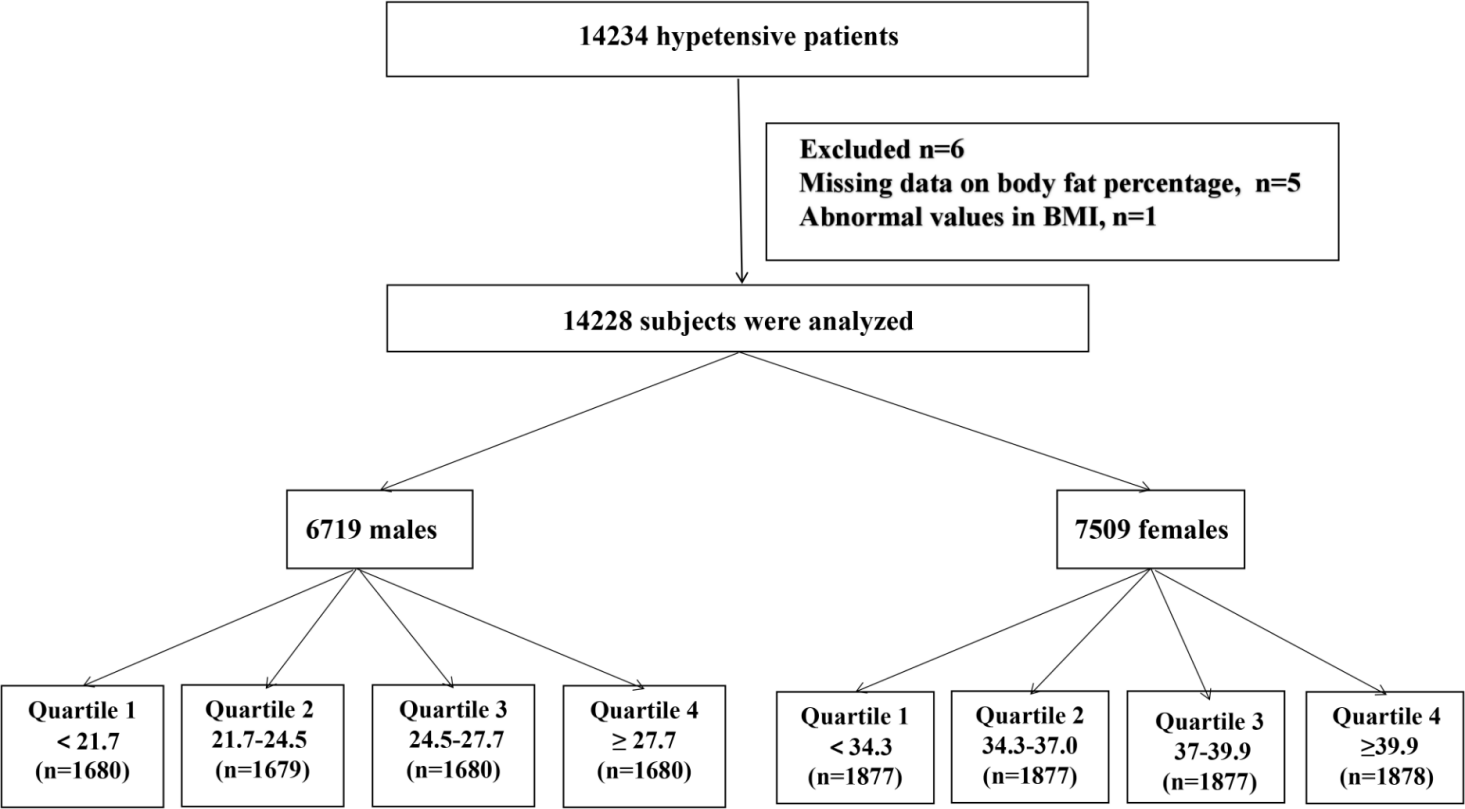
Flow chart of participants.

A total of 14234 patients with hypertension met the inclusion and exclusion criteria. After excluding patients with missing BFP data (n=5) and abnormal BMI values (n=1), 14228 subjects were included in the final analysis (**Figure 1**)

### Clinical characteristics

Demographic characteristics (gender and age), lifestyle variances (smoking status, drinking status, and physical activity), and medical history (diabetes, heart failure, hyperlipidemia, hypertension, and medication use) were gathered by trained specialized researchers. Blood pressure (BP) was measured using automatic electronic equipment (Omron; Dalian, China). Following a 10-minute rest, both systolic blood pressure (SBP) and diastolic blood pressure (DBP) were recorded, and the average values from three blood pressure measurements were documented.

Fasting venous blood samples were collected from all subjects. Subsequently, the blood samples were frozen and dispatched to the Shenzhen Biaojia Biotechnology Laboratory for analysis. Fasting blood glucose (FPG) and fasting blood lipids, including total cholesterol (TC), high-density lipoprotein cholesterol (HDL-C), low-density lipoprotein cholesterol (LDL-C), and triglycerides (TG), were analyzed using the Beckman Coulter automatic clinical analyzer. The homocysteine (Hcy) was measured using automatic clinical analyzers (Beckman Coulter, USA). The glomerular filtration rate (eGFR) was estimated using the Chronic Kidney Disease Epidemiology Collaboration (CKD-EPI) equation.

### Body composition assessment

The height, weight, and waist circumference (WC) of the study subjects were measured by trained researchers using standardized equipment. BMI was calculated by dividing the weight (in kilograms) by the square of the height (in meters). BFP was estimated using the Clínica University de Navarra-Body Adiposity Estimator (CUN-BAE) equation: BFP = -44.988 + (0.503 × age) + (10.689 × sex) + (3.172 × BMI) - (0.026 × BMI^2^) + (0.181 × BMI × sex) - (0.02 × BMI × age) - (0.005 × BMI^2^ × sex) + (0.00021 × BMI^2^ × age) (20). In this equation, a male gender is represented as 0, while a female gender is represented as 1. This formula has been utilized in various clinical research studies.

Since the recommended reference range of BFP for the Chinese population is currently unavailable, we will categorize the research subjects based on gender. They were divided into four groups according to BFP quartiles for each gender. For men, the BFP quartiles were defined as follows: below 21.7% (quartile 1), 21.7% - 24.5% (quartile 2), 24.5% - 27.2% (quartile 3), and 27.2% and above (quartile 4). In women, the BFP quartiles were: below 34.3% (quartile 1), 34.3% - 37.0% (quartile 2), 37.0% - 39.9% (quartile 3), and 39.9% and above (quartile 4)

### Definition of diabetes and blood glucose measurement

We used the Beckman Coulter automatic clinical analyzer to measure the subjects’ fasting blood glucose level. Diabetes in this study was defined as self-reported previous diagnosis of diabetes or fasting blood glucose ≥ 7.0 mmol/L and use of glucose-lowering drugs.

### Statistical analysis

For normally distributed continuous variables, data are typically presented as mean ± standard deviation (SD). In contrast, for continuous variables with a skewed distribution, data are usually presented as median (25th-75th percentile), while categorical variables are often depicted as count (%). In this study, a descriptive analysis was conducted based on the quartiles of BFP. The differences between gender groups were compared using either an ANOVA test or a chi-square test. The dose-response relationship between BFP and the risk of diabetes was assessed using a generalized additive model (GAM) and adjusted penalized spline method. A Multivariate logistic regression model, including odds ratio (OR) and 95%CI confidence interval, was utilized to control for the main covariates across three models and establish an independent association between BFP and diabetes risk. The models were as follows: Model 1: Crude model; Model 2: adjusted for age, physical activity, current smoking, and current drinking; Model 3: adjusted for age, SBP, DBP, current smoking, current drinking, physical activity, Hcy, TG, HDL-C, LDL-C, and eGFR, stroke, coronary heart disease (CHD), antihypertensive drugs, lipoprotein-lowering drugs. Furthermore, the potential interaction between BFP and diabetes was examined through stratified analysis and an interaction test.

All data analyses were conducted using the statistical package R (http://www.r-project.org) and Empower(R) (www.empowerstats.com; X&Y Solutions, Inc., Boston, MA). A two-tailed *P* < 0.05 was considered statistically significant.

## Result

### Baseline characteristics

A total of 6,719 male and 7,509 female hypertensive patients were included in the final analysis. The findings presented in **Table 1** reveal that men with higher BFP tend to be younger, non-smokers, have elevated levels of DBP, TC, TG, LDL-C, a higher prevalence of diabetes, and lower levels of HDL-C. Conversely, there was no significant statistical difference observed in current drinking habits and levels of SBP and eGFR. On the other hand, among women, the results slightly differ, as individuals with higher BFP are older and have lower levels of eGFR.

### Association of BFP with diabetes

**Figure 2:**
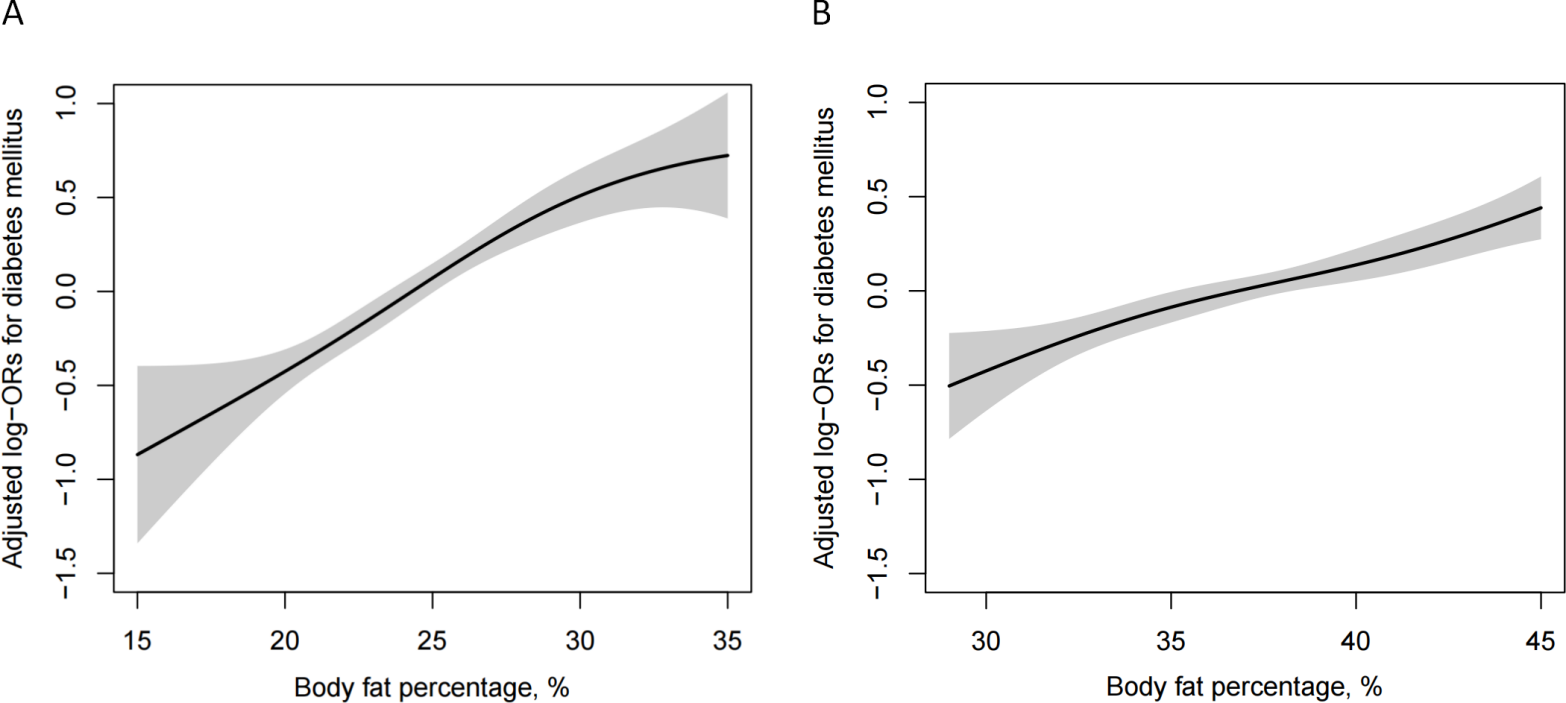
Dose-response relationship between body fat percentage and diabetes among men **(A)** and women **(B)**. Models were adjusted for age, SBP, DBP, current smoking, current drinking, physical activity, Hcy, TG, HDL-C, LDL-C, and eGFR. stroke, CHD, antihypertensive agents, lipid-lowering agents.

As shown in **Figure 2**, BFP was significantly positively associated with the risk of diabetes for both men and women. **Table 2** indicates that there is a dose-dependent relationship between BFP and diabetes risk. Regardless of whether the confounding factors were adjusted for, BFP of different genders was positively associated with the risk of diabetes (*P <*0.001). With BFP as the continuous variable, the risk of diabetes increased by 9% in the fully adjusted model in men (OR 1.09, 95% CI 1.07, 1.11), and the risk increased by 6% (OR 1.06, 95% CI 1.04, 1.07) in women. BFP was further divided into quartiles, in the men fully adjusted model, compared with Q1 (<21.7%), the risk of diabetes increased by 51% (OR 1.51, 95% CI 1.17, 1.94) in Q2 (21.7% -24.5%), 94% (OR 1.94, 95% CI 1.51, 2.49) in Q3 (24.5% -27.2%) and 176% (OR 2.76, 95% CI 2.15, 3.55) in Q4 (≥ 27.2%). In the fully adjusted women quartile model, compared with Q1 (<34.3%), there was no significant difference in the risk of diabetes in Q2 (34.3% -37.0%) group (OR 1.20, 95% CI 0.98, 1.47), while the risk in Q3 (37.0% -39.9%) increased by 57% (OR 1.57, 95% CI 1.29, 1.91), and 66% (OR 1.66, 95% CI 1.36, 2.03) in Q4 (≥ 39.9%). Although the positive association between BFP and diabetes is stable, there is still gender difference. Whether using BFP as a continuous variable (*P* = 0.016 for interaction) or a categorical variable in the quartile (*P* = 0.008 for interaction).

**Table 2:** Association between body fat percentage and diabetes mellitus among men and women.

Body fat percentage, %	N	Events, n (%)	Model 1		Model 2		Model 3	
			OR (95%CI)	*P* value	OR (95%CI)	*P* value	OR (95%CI)	*P* value
Men								
Per 1 unit increase	6719	1078 (16.0)	1.12 (1.10, 1.14)	<0.001	1.11 (1.09, 1.13)	<0.001	1.09 (1.07, 1.11)	<0.001
Quartiles								
Q1 (<21.7)	1680	132 (7.9)	Reference		Reference		Reference	
Q2 (21.7-24.5)	1679	221 (13.2)	1.78 (1.42, 2.23)	<0.001	1.70 (1.33, 2.17)	<0.001	1.51 (1.17, 1.94)	<0.001
Q3 (24.5-27.2)	1680	295 (17.6)	2.50 (2.01, 3.11)	<0.001	2.31 (1.83, 2.93)	<0.001	1.94 (1.51, 2.49)	<0.001
Q4 (≥27.2)	1680	430 (25.6)	4.03 (3.27, 4.97)	<0.001	3.56 (2.83, 4.46)	<0.001	2.76 (2.15, 3.55)	<0.001
P for trend				<0.001		<0.001		<0.001
Women								
Per 1 unit increase	7509	1539 (20.5)	1.08 (1.06, 1.09)	<0.001	1.08 (1.06, 1.09)	<0.001	1.06 (1.04, 1.07)	<0.001
Quartiles								
Q1 (<34.3)	1877	270 (14.4)	Reference		Reference		Reference	
Q2 (34.3-37.0)	1877	346 (18.4)	1.35 (1.13, 1.60)	0.001	1.34 (1.10, 1.63)	0.003	1.20 (0.98, 1.47)	0.074
Q3 (37.0-39.9)	1877	432 (23.0)	1.78 (1.50, 2.10)	<0.001	1.86 (1.54, 2.24)	<0.001	1.57 (1.29, 1.91)	<0.001
Q4 (≥39.9)	1878	491 (26.1)	2.11 (1.79, 2.48)	<0.001	2.09 (1.73, 2.51)	<0.001	1.66 (1.36, 2.03)	<0.001
*P* for trend				<0.001		<0.001		<0.001
*P* for interaction^a^				<0.001		0.009		0.016
*P* for interaction^b^				<0.001		0.003		0.008

Model 1: crude model.

Model 2: adjusted for age, physical activity, current smoking, current drinking.

Model 3: adjusted for age, SBP, DBP, current smoking, current drinking, physical activity, Hcy, TG, HDL-C, LDL-C, and eGFR. stroke, CHD, antihypertensive agents, lipid-lowering agents.

a *P* for interaction test: 2-way interaction of BFP (continuous) and sex on diabetes mellitus.

b *P* for interaction test: 2-way interaction of BFP (quartiles) and sex on diabetes mellitus.

### Subgroup analysis

**Figure 3:**
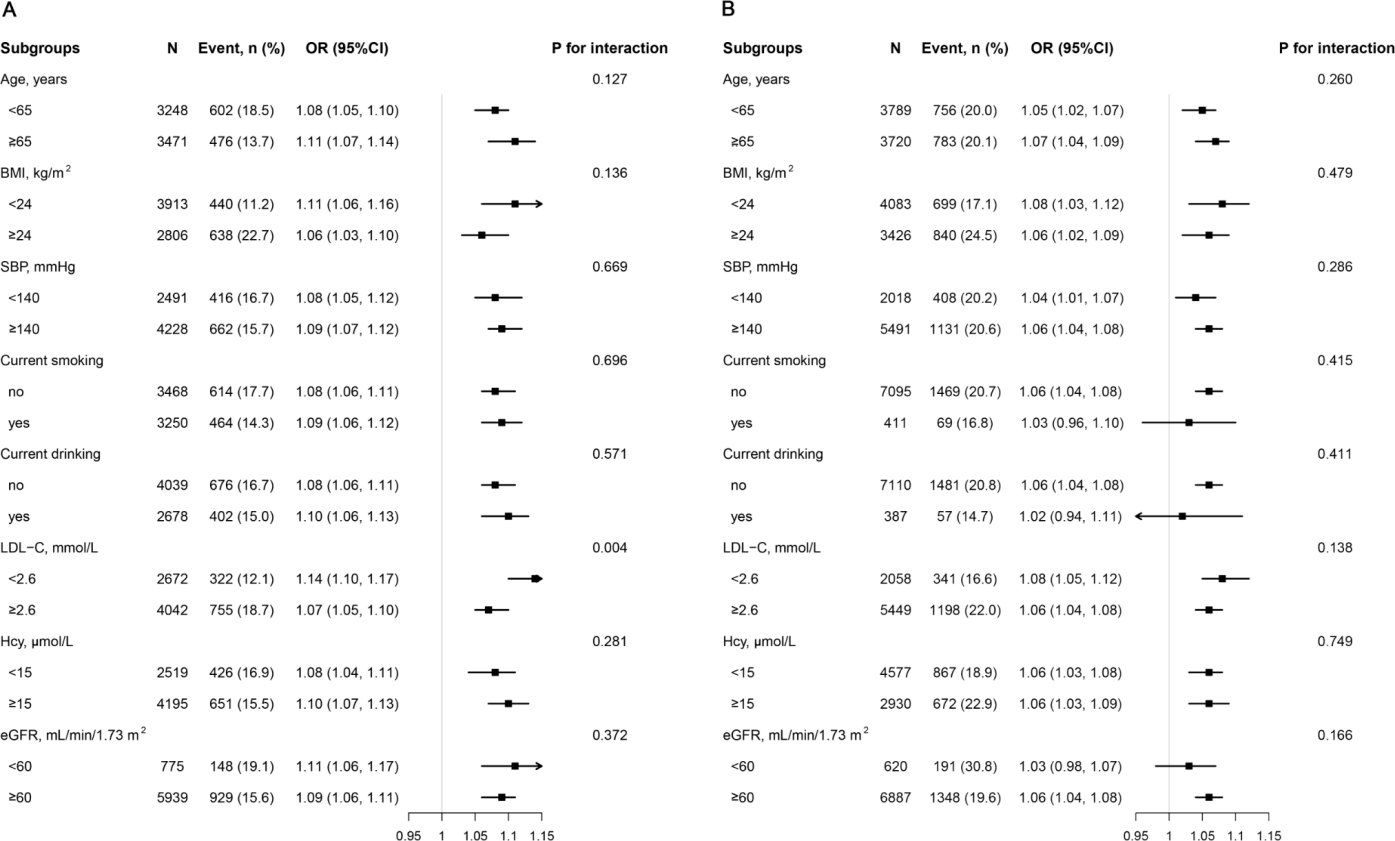
Subgroup analyses of the effect of BFP on diabetes among men **(A)** and women **(B)**. Each subgroup analysis adjusted, if not stratified, for age, SBP, DBP, current smoking, current drinking, physical activity, Hcy, TG, HDL-C, LDL-C, and eGFR. stroke, CHD, antihypertensive agents, lipid-lowering agents.

We conducted an analysis to investigate the relationship between BFP and diabetes within various subgroups (**Figure 3**), including age, BMI, SBP, current smoking, current drinking, LDL-C, Hcy, and eGFR. Our findings revealed a consistent positive association between BFP and diabetes across all stratified subgroups among women (with interaction *P* value > 0.05). Although the positive association between BFP and diabetes risk remained constant in men, it was more pronounced in individuals with lower LDL-C (OR 1.14, 95% CI 1.10, 1.17) levels compared to those with higher LDL-C (OR 1.07, 95% CI 1.05, 1.10) levels (*P* for interaction = 0.004).

## Discussion

This extensive cross-sectional study, encompassing a substantial sample size of 14,228 participants from the hypertensive population in southern China, revealed there is a positive association between BFP and diabetes. Even after adjusting for potential confounding variables, the association remained statistically significant. Notably, in men, this positive association was more pronounced. Additionally, the positive association between BFP and diabetes was consistent across all subgroups of women, and there is a more significant positive association between the two in men with LDL-C<2.6mmol/L.

There have been numerous speculations and reports on the pathogenesis of obesity and diabetes. As early as 2001, researchers discovered that adipocytes secrete resistin, which initially shed light on the link between obesity and insulin resistance (21). This discovery has since catalyzed the emergence of various research directions and disciplines, delving into mechanisms that lead to adipose tissue inflammation. These investigations include examining the role of adaptive immune cells and the activation and phenotype transformation of macrophages, all of which may contribute to insulin resistance (22).

The accumulation of fat is an important link in the physical damage caused by obesity. BFP has been preliminarily explored as an emerging indicator for evaluating body fat. Previous studies have investigated the connection between BFP and diabetes in various populations. Chen et al.’s study on 3367 Chinese subjects demonstrated a positive association between BFP and diabetes, highlighting BFP as an independent risk factor and a valuable diagnostic indicator for type 2 diabetes (23). Similarly, Peña J et al. found in a study involving 1920 Mexican participants that BFP is a key risk factor for type 2 diabetes, surpassing BMI in predictive value (24). Moreover, a prospective study with 1532 participants revealed a significant positive association between male trunk fat percentage and diabetes incidence, while the association with female trunk fat percentage, although positive, was not statistically significant (25). But as is well known that hypertension is an independent risk factor for diabetes often co-existing with the condition (26), is linked to insulin resistance in hypertensive individuals and serves as a confounding variable associated with overweight and obesity (27–29). High blood pressure can increase the risk of vascular complications in diabetic patients that emphasizing the importance of identifying high-risk hypertensive individuals with diabetes for prevention and management (30, 31). While previous studies have explored various other anthropometric indices for predicting diabetes in hypertensive populations (32), but our study uniquely focuses on the relationship between BFP and diabetes among hypertensive patients and the gender differences within it.

In the elderly population, a distinct gender-specific pattern in BFP distribution is observed. Younger male individuals tend to exhibit higher BFP values, whereas older female individuals demonstrate an opposite trend. This phenomenon can be attributed to age-related hormonal changes: in aging males, declining testosterone levels contribute to alterations in fat distribution patterns and progressive loss of muscle mass, consequently leading to reduced body fat percentage. Conversely, postmenopausal women experience significant estrogen depletion, which promotes visceral fat accumulation in abdominal regions, ultimately resulting in increased BFP. Our research also indicates a stronger positive association between BFP and diabetes in men compared to women. This disparity may stem from differences in male and female hormones or variations in fat distribution (33). Men are more prone to abdominal obesity and higher levels of visceral fat, while women typically accumulate fat in the buttocks and thighs, with a greater proportion of subcutaneous fat (34). Consequently, weight gain in men often results from increased visceral fat, which may significantly elevate the risk of diabetes. Research has found that fat in gluteus–femoral area in overweight and obese women has a protective effect on glucose and lipid related cardiac metabolic risks. Compared with subjects with widespread distribution of visceral fat, hip and thigh fat are associated with a beneficial fat factor profile and fewer pro-inflammatory molecules (35, 36). This distribution may offer protective effects against glucose and fat-related cardiac metabolic diseases (37). The study also confirmed that people with type 2 diabetes respond less to obesity treatment than those without type 2 diabetes, especially males. Women respond better to treatment than men (38). This could explain the heightened association between BFP and diabetes in men. Our findings also suggest that in men with lower LDL-C levels, the positive association between BFP and diabetes is more pronounced. Traditional treatments often involve lowering LDL-C to less than 2.6 mmol/L, or even lower, particularly for patients with known cardiovascular diseases or at high risk (39). However, intensive statin therapy may elevate the risk of developing diabetes (40, 41). Studies propose that patients with lower LDL-C levels may become complacent, adopt poorer lifestyles, gain weight, and subsequently develop diabetes. Additionally, impaired insulin secretion and intensified insulin resistance may contribute to this phenomenon (42).

This cross-sectional study, offers insights into the gender differences within the association between BFP and diabetes among hypertensive patients that less-explored. The study’s emphasis on a homogeneous population of hypertensive patients enhances result reliability, and detailed subgroup analyses contribute to result stability. However, this article also has certain limitations. Dual energy X-ray absorption or bioelectrical impedance analysis is considered the gold standard for measuring BFP (43). The formula we use to calculate BFP has been applied in multiple articles and is easier to obtain in clinical practice. But it still cannot achieve the same status as the gold standard (44). And the hypertensive participants enrolled in our study were not stratified according to the etiology of hypertension, including both primary and secondary hypertension cases. Secondly, this study is a cross-sectional study, which means that the results could not establish a causal relationship between BFP and diabetes. Despite performing multivariate corrections, it remains challenging to eliminate potential confounding factors. And we did not gather more detailed information about subjects, such as dietary structure. As a result, we cannot ascertain whether these factors have a regulatory effect on the association between BFP and the risk of diabetes. Lastly, this study focuses solely on the hypertensive population in southern China, raising uncertainties about the generalizability of the conclusions to other populations.

## Conclusion

BFP shows a positive association with the risk of diabetes among hypertensive patients in southern China. This association is notably stronger in men compared to women. It is recommended that men should pay more attention to changes in BFP than women. Further longitudinal studies are required to unveil the underlying mechanisms and establish a causal relationship between the two variables.

## Data Availability

The raw data supporting the conclusions of this article will be made available by the authors, without undue reservation.
